# The Newtonian potential inhomogeneity problem: non-uniform eigenstrains in cylinders of non-elliptical cross section

**DOI:** 10.1007/s10665-017-9923-9

**Published:** 2017-07-31

**Authors:** Duncan Joyce, William J. Parnell

**Affiliations:** School of Mathematics, University of Manchester, Oxford Road, Manchester, M13 9PL UK

**Keywords:** Conductivity, Eigenstrain, Eshelby tensor, Inclusion, Inhomogeneity, Non-ellipsoidal, Potential problem

## Abstract

Understanding the fields that are set up in and around inhomogeneities is of great importance in order to predict the manner in which heterogeneous media behave when subjected to applied loads or other fields, e.g., magnetic, electric, thermal, etc. The classical inhomogeneity problem of an ellipsoid embedded in an unbounded host or matrix medium has long been studied but is perhaps most associated with the name of Eshelby due to his seminal work in 1957, where in the context of the linear elasticity problem, he showed that for imposed far fields that correspond to uniform strains, the strain field induced inside the ellipsoid is also uniform. In Eshelby’s language, this corresponds to requiring a uniform eigenstrain in order to account for the presence of the ellipsoidal inhomogeneity, and the so-called *Eshelby tensor* arises, which is also uniform for ellipsoids. Since then, the Eshelby tensor has been determined by many authors for inhomogeneities of various shapes, but almost always for the case of uniform eigenstrains. In many application areas in fact, the case of non-uniform eigenstrains is of more physical significance, particularly when the inhomogeneity is non-ellipsoidal. In this article, a method is introduced, which approximates the Eshelby tensor for a variety of shaped inhomogeneities in the case of more complex eigenstrains by employing local polynomial expansions of both the eigenstrain and the resulting Eshelby tensor, in the case of the potential problem in two dimensions.

## Introduction

For almost two centuries now, significant interest has been focused on the ability to predict fields arising inside and surrounding inhomogeneities embedded in otherwise uniform host materials. In particular, the isolated inhomogeneity problem where a single inhomogeneity resides inside a host medium that is considered to be of infinite extent, with conditions imposed in the far field, is a classical one. In 1826, Poisson studied the perturbed field due to an isolated ellipsoid in the context of the Newtonian potential problem [1]. It was shown that the induced electric (or magnetic) field inside the ellipsoid is uniform given a uniform electric polarisation (or magnetisation). In 1873, Maxwell derived explicit expressions for this field [2]. In 1931, Dive [3] and independently in 1932 Nikliborc [4] proved that given an arbitrary imposed uniform electric polarisation in the far field, the electric polarisation induced inside an ellipsoid is also uniform. In the modern language of inhomogeneity problems, this is considered to be proof of the weak Eshelby conjecture for the Newtonian potential problem. This language itself arises following the seminal paper of Eshelby in 1957, who considered the isolated inhomogeneity problem in the case of linear elastostatics and proved that the induced strain in an ellipsoid is uniform given the arbitrary uniform strains in the far field [5]. In 1961, Eshelby conjectured that the ellipsoid is the only shape that has this property [6]. This being true for a uniform *arbitrary* far-field strain is known as the *weak* Eshelby conjecture. On the other hand, this being true for a uniform *specific* far-field strain is known as the *strong* Eshelby conjecture. Given the similarity of the Newtonian potential and linear elastostatics problems, differing mainly by their tensorial order, they are often discussed simultaneously [7], and hence the use of this term in both contexts. The strong conjecture in the context of the potential problem is true in two dimensions [8, 9], but it is *not* true in dimensions greater than two. This was illustrated in 2008 when Liu gave a non-ellipsoidal counterexample associated with a specific far-field loading [10].

For ellipsoidal inhomogeneity problems, the so-called *Eshelby tensor* arises naturally, as does its counterpart the *Hill tensor*. These can be shown to be uniform for homogeneous ellipsoidal inhomogeneities. These tensors arise specifically in the context of micromechanics and effective medium theory, with the aim of predicting the effective properties of inhomogeneous media [7, 11]. Speaking in the context of thermal conductivity as we do in this article, components of the Eshelby tensor written with respect to Cartesian coordinates in the isotropic case take the form:

1.1 $$\begin{aligned} \tilde{S}_{ij}&= {-}k_0 \frac{\partial ^2}{\partial \tilde{y}_i \partial \tilde{y}_j} \int _{\tilde{D}} \tilde{\mathcal {G}}(\tilde{{\mathbf {x}}}-\tilde{\mathbf {y}}) \mathrm{d}\tilde{{\mathbf {x}}}, \end{aligned}$$

where $k_0$ is the conductivity, $\tilde{D}$ is the ellipsoidal inhomogeneity, and $\tilde{\mathcal {G}}=1/(4\pi k_0|\tilde{{\mathbf {x}}}-\tilde{\mathbf {y}}|)$ is the associated Green’s tensor. Hill’s tensor $\mathbf {P}=k_0^{-1}\tilde{\mathbf {S}}.$ The associated two-dimensional problem is also frequently studied when $\tilde{D}$ is an ellipse, and $\tilde{\mathcal {G}}={-}1/(2\pi k_0)\log |\tilde{{\mathbf {x}}}-\tilde{\mathbf {y}}|.$ This tensor arises naturally *because* it is uniform for ellipsoidal inhomogeneities, and therefore, assuming uniform interior potential gradient ${\mathbf {e}}^1$, the associated integral equation gives (as will be shown below)

1.2 $$\begin{aligned} {\mathbf {e}}^1 = {\mathbf {e}}^{\infty } - (\kappa -1)\tilde{\mathbf {S}}{\mathbf {e}}^1, \quad \text {so that}\quad {\mathbf {e}}^1 = [\mathbf {I}+(\kappa -1)\tilde{\mathbf {S}}]^{-1}{\mathbf {e}}^{\infty }, \end{aligned}$$

where ${\mathbf {e}}^{\infty }$ is the uniform far-field potential gradient, and $\kappa =k_1/k_0$ where $k_1$ is the conductivity of the inhomogeneity.

For non-ellipsoidal inhomogeneities or in studies of, e.g. interacting inhomogeneities, in general, the temperature gradient will not be uniform, and therefore, the Eshelby tensor associated with non-ellipsoidal inhomogeneities is *not* generally a natural quantity to work with, since it will not arise naturally in the associated integral equation. In spite of this, the non-ellipsoidal problem has been studied frequently in the literature usually in connection with Eshelby’s conjecture, to prove that a tensor is *not* uniform for specific classes of inhomogeneities. Attention has been paid to polygonal and polyhedral inhomogeneities and the associated properties of Eshelby’s tensor [12–19]. The case of the so-called supersphere was considered in [20], which built on the work in [21–23]. For the case of the Newtonian potential problem and planar elastostatics, some analytical expressions have been derived for two-dimensional problems where inhomogeneities are either polygonal or their shape can be described by finite Laurent expansions [24, 25]. Further properties of the Eshelby tensor were deduced in [26, 27], including the relationship of the averaged Eshelby tensor for non-ellipsoidal inhomogeneities to their ellipsoidal counterparts.

The non-ellipsoidal problem is also considered since the so-called uniform *eigenstrains* can be considered in *inclusion* regions within a homogeneous host material. A non-zero eigenstrain field can be used to simulate the presence of an inhomogeneity inside a homogeneous host medium or can additionally be considered to incorporate some other physical effect, boundary presence, interacting inhomogeneities, or mismatch [28]. In order to simulate the presence of an isolated ellipsoidal inhomogeneity with uniform potential gradient imposed in the far field, the required eigenstrain is uniform [28]. Ru [29] developed a method based on conformal mappings to determine the strain field that arises in and around two-dimensional inhomogeneities of arbitrary cross section, due to uniform interior eigenstrain. Importantly, it should be noted that this is *not* equivalent to solving an inhomogeneity problem with uniform far-field loading because a uniform eigenstrain cannot account for the presence of an inhomogeneity of arbitrary cross section [28].

For the study of interior fields associated with non-ellipsoidal (non-elliptical in two dimensions) inhomogeneities, a more natural quantity to study is what we shall term here the *generalised Eshelby tensor* taking the form

1.3 $$\begin{aligned} \tilde{S}^*_{ij}(\tilde{{\mathbf {x}}})&= {-}k_0 \frac{\partial ^2}{\partial \tilde{x}_i \partial \tilde{x}_j} \int _{\tilde{D}}e^*(\tilde{\mathbf {y}}) \tilde{\mathcal {G}}(\tilde{{\mathbf {x}}}-\tilde{\mathbf {y}}) \mathrm{d}\tilde{\mathbf {y}}, \end{aligned}$$

where $e_*(\tilde{\mathbf {y}})$ is a *component* of the so-called *eigenstrain*. Note that the term *eigenstrain* is always used regardless of the application area. Rahman [30] derived explicit expressions for the polynomial eigenstrain problem associated with ellipsoidal inclusions in the elastostatics context. For arbitrary $\tilde{D}$ in the context of the thermal problem, if one considers polynomial eigenstrains, then the generalised Eshelby tensors take the form

1.4 $$\begin{aligned} \tilde{S}^{mnp}_{ij}(\tilde{{\mathbf {x}}})&= {-}k_0 \frac{\partial ^2}{\partial \tilde{x}_i \partial \tilde{x}_j} \int _{\tilde{D}}\left( \tilde{y}_1-\tilde{s}_1\right) ^m \left( \tilde{y}_2-\tilde{s}_2\right) ^n\left( \tilde{y}_3-\tilde{s}_3\right) ^p \tilde{\mathcal {G}}(\tilde{{\mathbf {x}}}-\tilde{\mathbf {y}}) \mathrm{d}\tilde{\mathbf {y}}, \end{aligned}$$

where for generality the polynomial is expanded about $\tilde{\mathbf {s}}.$ An interesting property for ellipsoids is that of *polynomial conservation* which states that Eshelby’s tensor will be a polynomial of order *N* when the eigenstrain is of order *N* [28, 30].

Regardless of the fact that a *generalised* Eshelby tensor of the form (1.3) or (1.4) is of more physical significance for non-ellipsoidal problems, such tensors do not appear to have been studied in the literature for general inhomogeneities $\tilde{D}.$ Mura ([28, Sect. 12]) states certain results associated with the polynomial conservation property which arise from results in the ellipsoidal potential theory context due to Ferrers [31] and Dyson [32]. For a more complete treatment of the inhomogeneous ellipsoid problem, see the more recent paper by Rahman [33]. Mura also uses these results in order to approximate the effect of non-uniformity of interior strain fields when two ellipsoidal inhomogeneities interact. However, even for isolated non-ellipsoidal cases, no results appear to have been derived except in the case when $N=0,$ the classical Eshelby tensor scenario. The case when $N\ge 1$ for non-ellipsoidal inhomogeneities is therefore the focus of the present article, and our attention is restricted to the two-dimensional case, i.e. when the generalised Eshelby tensor takes the form

1.5 $$\begin{aligned} \tilde{S}^{mn}_{ij}(\tilde{{\mathbf {x}}})&= {-}k_0 \frac{\partial ^2}{\partial \tilde{x}_i \partial \tilde{x}_j} \int _{\tilde{D}}\left( \tilde{y}_1-\tilde{s}_1\right) ^m \left( \tilde{y}_2-\tilde{s}_2\right) ^n \tilde{\mathcal {G}}(\tilde{{\mathbf {x}}}-\tilde{\mathbf {y}}) \mathrm{d}\tilde{\mathbf {y}}, \end{aligned}$$

with $\tilde{\mathcal {G}}={-}1/(2\pi k_0)\log |\tilde{{\mathbf {x}}}-\tilde{\mathbf {y}}|$ and where $\tilde{D}$ is *not* restricted to being elliptical.

Besides being of interest in their own right, the evaluation of such tensors would assist in developing approximate schemes for interacting inhomogeneity problems and in cases when eigenstrains are non-uniform. They may also be seen as approximations to cases when eigenstrains are non-polynomial, but Taylor series expansions are taken of the eigenstrains, expanded about locations inside $\tilde{D}.$

In this article, the focus is *not* in predicting internal fields within inhomogeneities, although this *is* clearly of interest and will be the focus of future work. Here polynomial approximations shall be determined for the generalised Eshelby tensors in the case of non-ellipsoidal inhomogeneities. In Sect.  2, we introduce the necessary mathematical background discussing both the inhomogeneity and inclusion problems and illustrating where Eshelby’s tensor arises in these contexts. Polynomial and more complex eigenstrain fields are then introduced in order to define the concept of a generalised Eshelby tensor. The method of approximating this tensor by via polynomial expansions is then described in Sect. 3. Application of the method to elliptical and non-elliptical domains is then described in Sect.  4. We summarise and discuss future directions in Sect.  5.

## Background

The motivation for the consideration of generalised Eshelby tensors is twofold. Firstly in the context of the prediction of induced fields associated with the presence of an isolated *inhomogeneity* (with different properties to those of the surrounding medium) embedded in an otherwise homogeneous medium, with some imposed condition at infinity. Secondly for the prediction of induced fields due to an isolated *inclusion* region (with the same properties as those of the homogeneous host medium) but within which a so-called *eigenstrain* is imposed.

Note the language used here, as introduced by Mura [28] which distinguishes an inclusion (same properties as those of the surrounding medium) from an inhomogeneity (different properties from those of the surrounding medium).

### Inhomogeneity problem

**Fig. 1:**
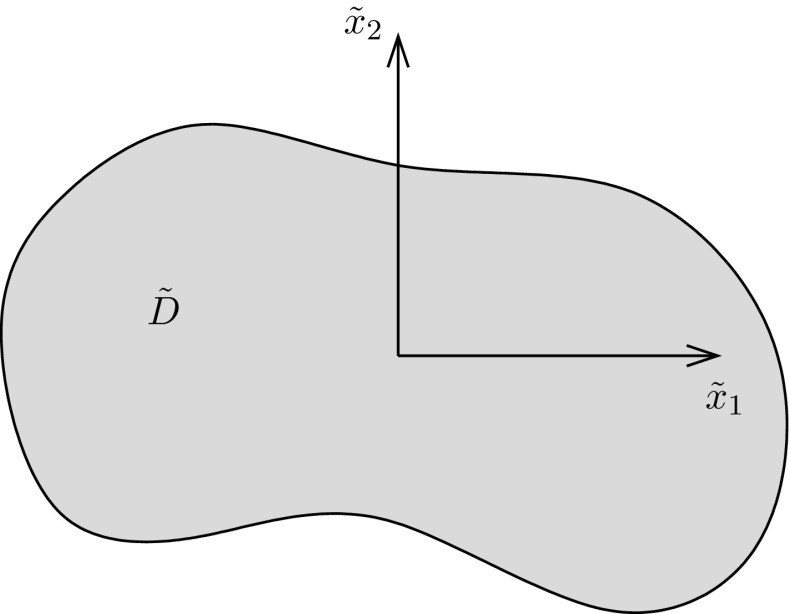
Configuration of the problem: a cylindrical inhomogeneity or inclusion region $\tilde{D}$ is embedded perfectly inside a host medium of infinite extent. The $\tilde{x}_1\tilde{x}_2$ plane is aligned with the cross section of the cylinder, which is denoted $\tilde{D}$

Consider an unbounded host medium of infinite extent into which a *cylindrical* inhomogeneity is embedded, which itself can be considered to be of infinite extent along its axis and place the $\tilde{x}_1\tilde{x}_2$ plane coincident with the cross section of the cylinder. With translational invariance in the $\tilde{x}_3$ direction then, the problem is purely two-dimensional, as depicted in Fig. 1 where the cross section of the inhomogeneity is defined as $\tilde{D}\in {\mathbb {R}}^2.$ Since it is often useful to consider a specific physical problem, certainly in terms of language and terminology, the potential problem here is described in the context of steady-state thermal conductivity.

The equation governing the steady-state temperature distribution $T(\tilde{{\mathbf {x}}})$ where $\tilde{{\mathbf {x}}}=(\tilde{x}_1,\,\tilde{x}_2)$ is the Cartesian position vector in the medium described above and depicted in Fig. 1 is

2.1 $$\begin{aligned} \frac{\partial {}}{\partial {\tilde{x}_i}}\left( C_{ij}(\tilde{{\mathbf {x}}})\frac{\partial {T}}{\partial {\tilde{x}_j}}\right) = 0, \quad i,\,j=1,\,2, \end{aligned}$$

where the assumption is that no heat sources are present. The free-space Green’s function associated with the *host* phase satisfies

2.2 $$\begin{aligned} \frac{\partial {}}{\partial {\tilde{x}_i}}\left( C_{ij}^0\frac{\partial {\tilde{\mathcal {G}}}}{\partial {\tilde{x}_j}}(\tilde{{\mathbf {x}}}-\tilde{\mathbf {y}})\right) + \delta (\tilde{{\mathbf {x}}}-\tilde{\mathbf {y}})&= 0. \end{aligned}$$

Assuming continuity of temperature and normal flux across $\partial \tilde{D},$ the resulting temperature distribution may be straightforwardly derived in integral equation form as

2.3 $$\begin{aligned} T(\tilde{{\mathbf {x}}}) = T^{\infty }(\tilde{{\mathbf {x}}}) - \left( C_{kj}^1-C_{kj}^0\right) \int _{\tilde{D}}\frac{\partial {T}}{\partial {\tilde{y}_k}} (\tilde{\mathbf {y}})\frac{\partial {\tilde{\mathcal {G}}}}{\partial {\tilde{y}_j}} (\tilde{{\mathbf {x}}}-\tilde{\mathbf {y}}) \mathrm{d}\tilde{\mathbf {y}}, \end{aligned}$$

which holds for all $\tilde{{\mathbf {x}}}.$ Here $T^{\infty }(\tilde{{\mathbf {x}}})$ is the solution to the equivalent problem satisfying (2.1) with no inhomogeneity present (or equivalently with $C_{ij}^1 = C_{ij}^0$). Upon taking derivatives of (2.3) with respect to $\tilde{x}_i$ and noting the property $\partial \tilde{\mathcal {G}}/\partial \tilde{y}_i= {-}\partial \tilde{\mathcal {G}}/\partial \tilde{x}_i$ it is found that for all $\tilde{{\mathbf {x}}},$

2.4 $$\begin{aligned} e_i(\tilde{{\mathbf {x}}})&= e_i^{\infty }(\tilde{{\mathbf {x}}}) + \left( C_{kj}^1-C_{kj}^0\right) \frac{\partial ^{2}{}}{\partial {\tilde{x}_i}\partial {\tilde{x}_j}}\int _{\tilde{D}}e_k(\tilde{\mathbf {y}})\tilde{\mathcal {G}}(\tilde{{\mathbf {x}}}-\tilde{\mathbf {y}}) \mathrm{d}\tilde{\mathbf {y}}, \end{aligned}$$

where the *i*th component of the temperature gradient has been defined as $e_i=\partial T/\partial \tilde{x}_i.$

If attention is restricted to anisotropy where the principal axes of the host and inhomogeneity are aligned, all anisotropic potential problem inhomogeneity problems can be reduced to isotropic ones by rescaling the spatial variable $\tilde{{\mathbf {x}}}$ which modifies the inhomogeneity shape. The problem is then solved in the scaled domain and mapped back to physical space [7]. Interest here is for general shaped inhomogeneities, and therefore we consider the isotropic problem only. Therefore take $C^0_{ij}=k_0\delta _{ij},\, C^1_{ij}=k_1\delta _{ij}$ and (2.4) becomes

2.5 $$\begin{aligned} e_i(\tilde{{\mathbf {x}}})&= e_i^{\infty }(\tilde{{\mathbf {x}}}) + (\kappa -1)\frac{\partial ^{2}{}}{\partial {\tilde{x}_i}\partial {\tilde{x}_j}}\int _{\tilde{D}}e_j(\tilde{\mathbf {y}})\tilde{G}(\tilde{{\mathbf {x}}}-\tilde{\mathbf {y}}) \mathrm{d}\tilde{\mathbf {y}}, \end{aligned}$$

where $\kappa =k_1/k_0$ and we have introduced the associated two-dimensional isotropic Greens function $\tilde{G}$ satisfying $\nabla ^2 \tilde{G} + \delta (\tilde{{\mathbf {x}}}-\tilde{\mathbf {y}}) = 0,$ i.e.

2.6 $$\begin{aligned} \tilde{G}(\tilde{{\mathbf {x}}}-\tilde{\mathbf {y}})&= -\dfrac{1}{2\pi }\log |\tilde{{\mathbf {x}}}-\tilde{\mathbf {y}}|. \end{aligned}$$

It is important to note that different references place the Dirac delta function on either side of the governing equation for the Green’s function. Of course, this merely changes the sign of the Green’s function, but it is important in terms of lifting information directly from one reference to another. We follow the convention in [7, 28].

Equation (2.5) is an integral equation for the potential gradient ${\mathbf {e}}$ and determining this *inside* $\tilde{D},$ which we shall denote as ${\mathbf {e}}^1$ then tells us the field everywhere. If one assumes that ${\mathbf {e}}^{\infty }$ is a uniform vector, taking $\mathbf {y}\in \tilde{D}$ and assume that ${\mathbf {e}}^1$ is also uniform, we arrive at the expression

2.7 $$\begin{aligned} {\mathbf {e}}^1&= \varvec{\alpha } - (\kappa -1)\tilde{\mathbf {S}}{\mathbf {e}}^1. \end{aligned}$$

This is simply (1.2), where $\tilde{\mathbf {S}}$ is Eshelby’s tensor with components

2.8 $$\begin{aligned} \tilde{S}_{ij}&= {-}\frac{\partial ^{2}{}}{\partial {\tilde{x}_i}\partial {\tilde{x}_j}}\int _{\tilde{D}}\tilde{G}(\tilde{{\mathbf {x}}}-\tilde{\mathbf {y}}) \mathrm{d}\mathbf {y}, \end{aligned}$$

and where deriving (2.7) has exploited the symmetry $S_{ij}=S_{ji}.$ Under these assumptions of uniformity, Eq. (2.7) is then only consistent if $\tilde{\mathbf {S}}$ is a uniform tensor, which, from Eshelby’s strong conjecture in two dimensions, we know *is only true in the case of elliptical inhomogeneities* $\tilde{D}.$ In two dimensions then, when the far-field temperature gradient if uniform, then one can use (2.7) in the case of isolated elliptical inhomogeneities to predict the uniform interior fields and Eshelby’s tensor takes the form ([7, (4.36)])

2.9 $$\begin{aligned} \tilde{S}_{ij}&= \frac{\epsilon }{1+\epsilon }\delta _{i1}\delta _{j1} + \frac{1}{1+\epsilon }\delta _{i2}\delta _{j2}, \end{aligned}$$

where $\epsilon =a_2/a_1$ where $a_j$ is the semi-axis of the ellipse directed along the $x_j$ axis. However, for non-elliptical inhomogeneities one must return to (2.5) and solve this integral equation. This approach also applies even for elliptical inhomogeneities when the far-field temperature gradient is non-uniform! One approach would be to *pose* some form for the interior strain in some basis, e.g. a polynomial expansion, e.g. for $\tilde{{\mathbf {x}}}_1\in \tilde{D},$

2.10 $$\begin{aligned} e_j(\tilde{\mathbf {y}}) = \sum _{m=0}^M\sum _{n=0}^N A^j_{mn} \left( \tilde{y}_1-\tilde{s}_1\right) ^m \left( \tilde{y}_2-\tilde{s}_2\right) ^n,\quad j=1,\,2, \end{aligned}$$

for some coefficients $A^j_{mn},\, j=1,\,2$ to be determined. Inserting these expansions in the integral equation will lead to consistency conditions on the coefficients (a linear system) with coefficients that are integrals of the form

2.11 $$\begin{aligned} \tilde{S}^{mn}_{ij}(\tilde{{\mathbf {x}}})&= {-}\frac{\partial ^{2}{}}{\partial {\tilde{x}_i}\partial {\tilde{x}_j}}\int _{\tilde{D}}^{}{\left( \tilde{y}_1-\tilde{s}_1\right) ^m \left( \tilde{y}_2-\tilde{s}_2\right) ^n \tilde{G}(\tilde{{\mathbf {x}}}-\tilde{\mathbf {y}})}d{\tilde{\mathbf {y}}}. \end{aligned}$$

Clearly such an expansion (2.10) could not be expected to converge everywhere but can certainly be of use. This method has been utilised to predict the fields interior to interacting ellipsoids in [28, Sect. 23] for example.

### Inclusion problem

Consider now a different configuration to the previous section. An unbounded host medium of infinite extent contains within it an *inclusion*—a *cylindrical* region which is considered to be of infinite extent along its axis and place the $\tilde{x}_1\tilde{x}_2$ plane coincident with the cross section of the cylinder. The conductivity tensor of the inclusion region is the same as its exterior. So-called *eigenstrains* (independent of $\tilde{x}_3$) are imposed in this region which induce a perturbed field. Note that unlike the previous section there is no imposed field at infinity. As with the previous section, with translational invariance in the $\tilde{x}_3$ direction then, the problem is purely two-dimensional. Following Mura [28] but in the potential context, the equation governing *T* subject to the eigenstrain field ${\mathbf {e}}^*$ is

2.12 $$\begin{aligned} \frac{\partial {}}{\partial {\tilde{x}_i}}\left( C_{ij}^0\frac{\partial {T}}{\partial {\tilde{x}_j}}\right) = \frac{\partial {}}{\partial {\tilde{x}_i}}\left( \chi (\tilde{{\mathbf {x}}})C_{ij}^0e^*_j(\tilde{{\mathbf {x}}})\right) ,\quad i,\,j=1,\,2, \end{aligned}$$

where $\chi (\tilde{{\mathbf {x}}})=1$ when $\tilde{{\mathbf {x}}}\in \tilde{D}$ and is zero otherwise. Using the Green’s function as defined in (2.2) the induced field can be written

2.13 $$\begin{aligned} T(\tilde{{\mathbf {x}}}) = {-}C_{kj}^0\int _{\tilde{D}}e_k^*(\tilde{\mathbf {y}})\frac{\partial {\tilde{\mathcal {G}}}}{\partial {\tilde{y}_j}}(\tilde{{\mathbf {x}}}-\tilde{\mathbf {y}}) \mathrm{d}\tilde{\mathbf {y}}, \end{aligned}$$

which holds for all $\tilde{{\mathbf {x}}}.$ As in the previous section, taking derivatives of (2.3) with respect to $\tilde{x}_i$ and noting the property $\partial \tilde{\mathcal {G}}/\partial \tilde{y}_i= {-}\partial \tilde{\mathcal {G}}/\partial \tilde{x}_i$ it is found that for all $\tilde{{\mathbf {x}}},$

2.14 $$\begin{aligned} e_i(\tilde{{\mathbf {x}}})&= C_{jk}^0\frac{\partial ^{2}{}}{\partial {\tilde{x}_i}\partial {\tilde{x}_j}}\int _{\tilde{D}}e_k^*(\tilde{\mathbf {y}})\tilde{\mathcal {G}}(\tilde{{\mathbf {x}}}-\tilde{\mathbf {y}}) \mathrm{d}\tilde{\mathbf {y}}. \end{aligned}$$

Following the same arguments as for the inhomogeneity problem if attention is restricted to anisotropy where the principal axes of the host and inhomogeneity are aligned, all anisotropic problems for general $\tilde{D}$ reduce to the isotropic case due to scaling arguments and therefore set $C^0_{ij}=k_0\delta _{ij}$ so that (2.14) becomes

2.15 $$\begin{aligned} e_i(\tilde{{\mathbf {x}}})&= \frac{\partial ^{2}{}}{\partial {\tilde{x}_i}\partial {\tilde{x}_j}}\int _{\tilde{D}}e_j^*(\tilde{\mathbf {y}})\tilde{G}(\tilde{{\mathbf {x}}}-\tilde{\mathbf {y}}) \mathrm{d}\tilde{\mathbf {y}}, \end{aligned}$$

where $\tilde{G}$ is defined in (2.6). With eigenstrain components $e_j^*$ in the form of polynomials, the right-hand side of (2.15) can clearly then be written as linear combinations of terms in the form (2.11).

One can employ eigenstrain to ‘represent’ the effects of an inhomogeneity [28]. First add some far-field forcing to the problem so that (2.15) becomes

2.16 $$\begin{aligned} e_i(\tilde{{\mathbf {x}}})&= e^{\infty }_i(\tilde{{\mathbf {x}}}) + \frac{\partial ^{2}{}}{\partial {\tilde{x}_i}\partial {\tilde{x}_j}}\int _{\tilde{D}}e_j^*(\tilde{\mathbf {y}})\tilde{G}(\tilde{{\mathbf {x}}}-\tilde{\mathbf {y}}) \mathrm{d}\tilde{\mathbf {y}}. \end{aligned}$$

Now comparing (2.16) with (2.5), we see that these are equivalent if

2.17 $$\begin{aligned} e_j^*(\tilde{{\mathbf {x}}})&= (\kappa -1)e_j(\tilde{{\mathbf {x}}}). \end{aligned}$$

In the case of an elliptical inhomogeneity with uniform $e_i^{\infty },\, \tilde{S}_{ij}$ is uniform and is given by (2.9), and we have

2.18 $$\begin{aligned} e_i^*&= (\kappa -1){\mathcal {A}}_{ij} e_j^{\infty }, \end{aligned}$$

where $\varvec{{\mathcal {A}}}=[\mathbf {I}+(\kappa -1)\tilde{\mathbf {S}}]^{-1}$ is known as the *concentration tensor* [7]. For imposed fields that are of polynomial form a polynomial eigenstrain of the same order can be employed to represent an inhomogeneity [28]. For more complex shaped domains, however, a general eigenstrain would be required.

### Generalised Eshelby tensors

Let us call (2.11) the generalised Eshelby tensor of order $(m,\,n).$ Upon defining

2.19 $$\begin{aligned} \tilde{J}^{mn}(\tilde{{\mathbf {x}}};\,\tilde{\mathbf {s}})&= {-}\int _{\tilde{D}} \left( \tilde{y}_1-\tilde{s}_1\right) ^m \left( \tilde{y}_2-\tilde{s}_2\right) ^n \tilde{G}(\tilde{{\mathbf {x}}}-\tilde{\mathbf {y}}) \mathrm{d}\tilde{\mathbf {y}}, \end{aligned}$$

we have

2.20 $$\begin{aligned} \tilde{S}_{ij}^{mn}(\tilde{{\mathbf {x}}})&= \frac{\partial ^{2}{\tilde{J}^{mn}(\tilde{{\mathbf {x}}};\,\tilde{\mathbf {s}})}}{\partial {\tilde{x}_i}\partial {\tilde{x}_j}}. \end{aligned}$$

Determining explicit results for these tensors for general $\tilde{D}$ is clearly difficult, if not impossible, unless we restrict attention to specific classes of $\tilde{D}.$ Instead, then consider an approach that is based on polynomial expansions. The general methodology shall be developed in the next section before it is applied to specific cases.

As we shall discuss in the Sect. 3, we will seek polynomial expansions as approximations to $\tilde{J}^{mn}$ for a given $\tilde{D}.$ As should be expected, it transpires that the choice of location about which the polynomial is expanded is extremely influential as to the domain of validity of the polynomial expansion. Suppose therefore that the polynomial expansion is centred on $\tilde{{\mathbf {x}}}=\tilde{{\mathbf {r}}}.$ The methodology to follow relies upon orthogonality and therefore $\tilde{J}^{mn}(\tilde{{\mathbf {x}}};\,\tilde{\mathbf {s}})$ shall first be written as a linear combination of $\tilde{J}^{mn}(\tilde{{\mathbf {x}}};\,\tilde{\mathbf {s}}),$ i.e. we write

2.21 $$\begin{aligned} \tilde{J}^{mn}(\tilde{{\mathbf {x}}};\,\tilde{\mathbf {s}})&= {-}\int _{\tilde{D}} \left( \tilde{y}_1-\tilde{r}_1+\tilde{r}_1- \tilde{s}_1\right) ^m \left( \tilde{y}_2-\tilde{r}_2+\tilde{r}_2 - \tilde{s}_2\right) ^n \tilde{G}(\tilde{{\mathbf {x}}}-\tilde{\mathbf {y}}) \mathrm{d}\tilde{\mathbf {y}} \end{aligned}$$

2.22 $$\begin{aligned}&= {-}\sum _{k=0}^m\sum _{\ell =0}^n \left( {\begin{array}{c}m\\ k\end{array}}\right) \left( {\begin{array}{c}n\\ \ell \end{array}}\right) \left( \tilde{r}_1 -\tilde{s}_1\right) ^k\left( \tilde{r}_2-\tilde{s}_2\right) ^{\ell } \int _{\tilde{D}}\left( \tilde{y}_1-\tilde{r}_1\right) ^k \left( \tilde{y}_2-\tilde{r}_2\right) ^{\ell } \tilde{G}(\tilde{{\mathbf {x}}}-\tilde{\mathbf {y}}) \mathrm{d}\tilde{\mathbf {y}} \end{aligned}$$

2.23 $$\begin{aligned}&= \sum _{k=0}^m\sum _{\ell =0}^n \left( {\begin{array}{c}m\\ k\end{array}}\right) \left( {\begin{array}{c}n\\ \ell \end{array}}\right) \left( \tilde{r}_1-\tilde{s}_1\right) ^k \left( \tilde{r}_2-\tilde{s}_2\right) ^{\ell }\tilde{J}^{k\ell }(\tilde{{\mathbf {x}}};\,\tilde{{\mathbf {r}}}). \end{aligned}$$

**Fig. 2:**
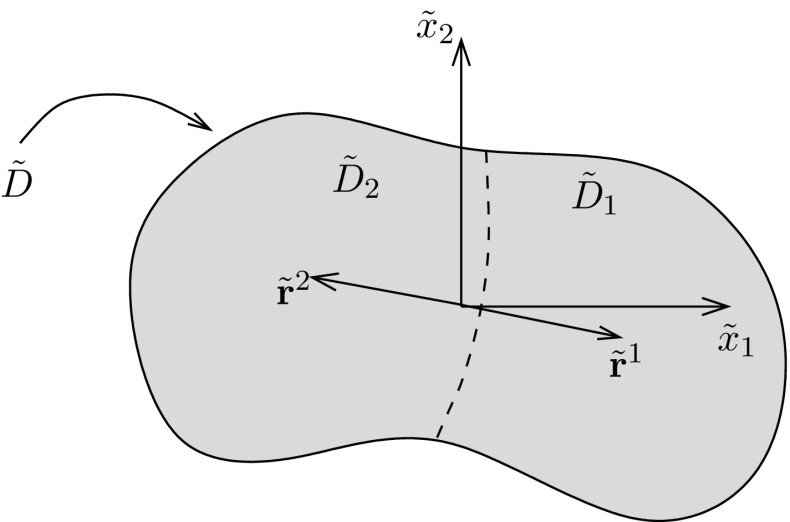
The region $\tilde{D}$ is split into two non-intersecting regions $\tilde{D}_1$ and $\tilde{D}_2,$ boundary of which here is depicted by the *dashed line*. A polynomial expansion approximation for the function $J^{mn}$ is sought locally in each region via an expansion about $\tilde{{\mathbf {x}}}=\tilde{{\mathbf {r}}}^j,$ where $j=1,\,2.$ An expression for the function $J^{mn}$ is then obtained by combining these local expansions via support functions $\chi ({\mathbf {x}})$

Further, in an effort to yield a polynomial expansion that is convergent over a larger domain, one can expand about distinct points $\tilde{{\mathbf {r}}}^j,\, j=1,\,2,\ldots ,p$ and then split the domain into non-intersecting support regions say $\tilde{D}_j$ (such that $\tilde{D}=\tilde{D}_1\cup \tilde{D}_2\cup \cdots \cup \tilde{D}_{p}$) over which the expansion is locally valid. For example, with reference to Fig. 2 write (2.23) as

2.24 $$\begin{aligned} \tilde{J}^{mn}(\tilde{{\mathbf {x}}};\,\tilde{\mathbf {s}})&= \sum _{j=1}^p\chi _j(\tilde{{\mathbf {x}}})\left( \sum _{k=0}^m\sum _{\ell =0}^n \left( {\begin{array}{c}m\\ k\end{array}}\right) \left( {\begin{array}{c}n\\ \ell \end{array}}\right) \left( \tilde{r}^j_1-\tilde{s}_1\right) ^k\left( \tilde{r}^j_2-\tilde{s}_2\right) ^{\ell }\tilde{J}^{k\ell }\left( \tilde{{\mathbf {x}}};\,\tilde{{\mathbf {r}}}^j\right) \right) , \end{aligned}$$

where

2.25 $$\begin{aligned} \chi (\tilde{{\mathbf {x}}}) =\left\{ \begin{array}{l@{\quad }l} 1, &{} \tilde{{\mathbf {x}}}\in \tilde{D}_j, \\ 0, &{} \tilde{{\mathbf {x}}}\notin \tilde{D}_j. \end{array}\right. \end{aligned}$$

### More complex eigenstrains

Note that in principal more complex eigenstrain fields can be considered in (2.15); particularly in the context of the local expansion approach (2.24). One would Taylor expand the eigenstrain about $\tilde{{\mathbf {r}}}^j,\, j=1,\,2,\ldots ,p$ inside $\tilde{D}$ as above, which leads to generalised Eshelby tensors (polynomial eigenstrains). For example, if we have

2.26 $$\begin{aligned} {\mathcal {I}}({\mathbf {x}})&= \int _{\tilde{D}}f(\tilde{\mathbf {y}}) G(\tilde{{\mathbf {x}}}-\tilde{\mathbf {y}}) \mathrm{d}\tilde{\mathbf {y}}, \end{aligned}$$

then in $\tilde{D}_j,$ Taylor expand $f(\tilde{\mathbf {y}})$ above $\tilde{\mathbf {y}}=\tilde{{\mathbf {r}}}$ to give

2.27 $$\begin{aligned} \tilde{{\mathcal {I}}}({\mathbf {x}})&= \sum _{m,n=0}^{\infty }\int _{\tilde{D}_j}\alpha _{mn}\left( \tilde{y}_1-\tilde{r}_1\right) ^m\left( \tilde{y}_2-\tilde{r}_2\right) ^n G(\tilde{{\mathbf {x}}}-\tilde{\mathbf {y}})\mathrm{d}\tilde{\mathbf {y}}, \end{aligned}$$

so that upon truncating

2.28 $$\begin{aligned} \tilde{{\mathcal {I}}}(\tilde{{\mathbf {x}}})\approx \tilde{{\mathcal {I}}}_{M,N}(\tilde{{\mathbf {x}}})&= \sum _{m,n=0}^{M,N}\int _{\tilde{D}_j}\alpha _{mn}\left( \tilde{y}_1-\tilde{r}_1\right) ^m\left( \tilde{y}_2-\tilde{r}_2\right) ^n G(\tilde{{\mathbf {x}}}-\tilde{\mathbf {y}}) \mathrm{d}\tilde{\mathbf {y}} \end{aligned}$$

2.29 $$\begin{aligned}&= \sum _{m,n=0}^{M,N}\left( -\alpha _{mn}\right) \tilde{J}^{mn}(\tilde{{\mathbf {x}}};\,\tilde{{\mathbf {r}}}). \end{aligned}$$

## Evaluation of the generalised Eshelby tensor for polynomial eigenstrains

**Fig. 3:**
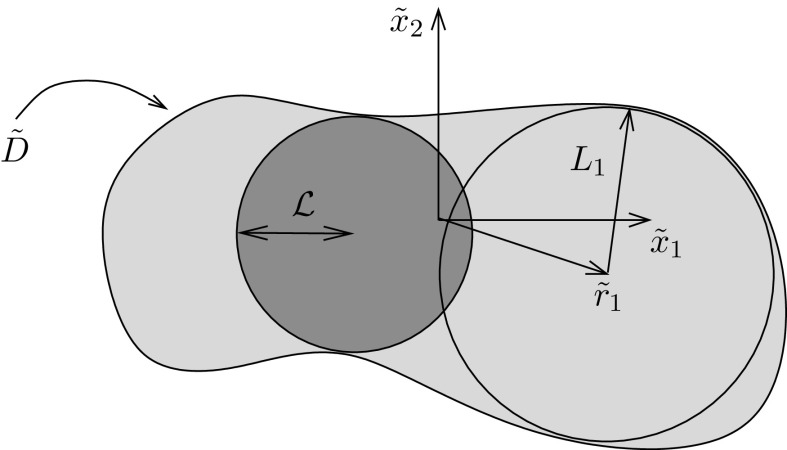
Depicting lengthscales of the inhomogeneity $\tilde{D}.$ The local expansion about $\tilde{{\mathbf {r}}}_1$ is scaled on $L_1$ for convenience. Once this local expansion is determined, in order to contribute to the general expansion scheme, we reintroduce $L_1.$ Finally, once the global expansion has been determined, this is scaled on some lengthscale, e.g. the radius ${\mathcal {L}}$ of the smallest circle that can be inscribed into $\tilde{D}$

Consider the situation where we need to determine $\tilde{J}(\tilde{{\mathbf {x}}};\,\tilde{{\mathbf {r}}}^j)$ as in (2.24), with reference to Fig.  2. As shall be explained below we will first re-scale these local coordinates and then seek polynomial expansions in the scaled coordinates ${\mathbf {x}}-{\mathbf {r}}^j$ for this local expansion. In order to use this expression in (2.24) one needs to re-introduce the scaling so that an expansion is determined in each local subregion $\tilde{D}_j$ terms of $\tilde{{\mathbf {x}}}-\tilde{{\mathbf {r}}}^j.$ Once a global expansion is determined it is then sensible to non-dimensionalise on some associated lengthscale of the inhomogeneity region ${\mathcal {L}},$ e.g. smallest circle that can be inscribed in the region whilst still touching the boundary at two points, as depicted in Fig. 3.

### Non-dimensionalisation

Since we are in two dimensions, we introduce the planar polar coordinate systems

3.1 $$\begin{aligned} \tilde{{\mathbf {x}}}-\tilde{{\mathbf {r}}}^j = \tilde{\rho }(\cos {\theta },\,\sin {\theta }),\quad \tilde{\mathbf {y}}-\tilde{{\mathbf {r}}}^j = \tilde{\zeta }(\cos \phi ,\,\sin \phi ), \end{aligned}$$

with $\tilde{\rho },\,\tilde{\zeta }\ge 0$ and $0\le \theta ,\,\phi <2\pi .$ Define the boundary of the inhomogeneity as $\tilde{\rho }=\tilde{f}_j(\theta )=L_jf_j(\theta ),$ where $L_j=\min \tilde{f}_j(\theta )$ so that $f_j(\theta )\ge 1,$ noting the subscript *j* that indicates dependence on ${\mathbf {r}}^j$ since this will affect the definition of $\tilde{f},\, f$ and $L_j.$ Finally, employ the scalings $\tilde{\rho }=L_j\rho ,\, \tilde{\zeta }=L_j\zeta ,$ and define

3.2 $$\begin{aligned} {\mathbf {x}}-{\mathbf {r}}^j = \rho (\cos {\theta },\,\sin {\theta }), \quad \mathbf {y}-{\mathbf {r}}^j = \zeta (\cos \phi ,\,\sin \phi ), \end{aligned}$$

and *D* as the scaled domain over which we integrate. We can write

3.3 $$\begin{aligned} \tilde{J}^{mn}\left( \tilde{{\mathbf {x}}};\,\tilde{{\mathbf {r}}}^j\right)&= \frac{L_j^{m+n+2}}{4\pi }\int _{0}^{2\pi }\int _0^{f_j(\theta )} \zeta ^{m+n+1}\cos ^{\delta }\phi \sin ^{\xi }\phi \ln L_j^2\left( \rho ^2+\zeta ^2-2\rho \zeta \cos (\theta -\phi )\right) \mathrm{d}\zeta \mathrm{d}\phi \end{aligned}$$

3.4 $$\begin{aligned}&= L_j^{m+n+2}\ln L_j^2 C_j + L_j^{m+n+2}J^{mn}\left( {\mathbf {x}};\,{\mathbf {r}}^j\right) , \end{aligned}$$

where

3.5 $$\begin{aligned}&C_j = \frac{1}{4\pi }\int _{0}^{2\pi }\int _0^{f_j(\theta )} \zeta ^{m+n+1}\cos ^{\delta }\phi \sin ^{\xi }\phi \mathrm{d}\zeta \mathrm{d}\phi , \end{aligned}$$

3.6 $$\begin{aligned}&J^{mn}\left( {\mathbf {x}};\,{\mathbf {r}}^j\right) = \frac{1}{4\pi }\int _{0}^{2\pi }\int _0^{f_j(\theta )} \zeta ^{m+n+1}\cos ^{\delta }\phi \sin ^{\xi }\phi \ln \left( \rho ^2+\zeta ^2-2\rho \zeta \cos (\theta -\phi )\right) \mathrm{d}\zeta \mathrm{d}\phi . \end{aligned}$$

### Polynomial approximations

For general *f* (i.e. for a general shaped inhomogeneity *D*), it is clearly not possible to obtain an analytical form for the expression (3.6); generally, it must be evaluated numerically for any point ${\mathbf {x}}\in D.$ Given its importance in a number of inhomogeneity and inclusion problems as indicated above, it would therefore be useful if *approximate* schemes can be developed in order to generate expressions for the right-hand side. Let us suppose that we may expand *J* in powers of its argument, e.g. in the form

3.7 $$\begin{aligned} J^{mn}\left( {\mathbf {x}};\,{\mathbf {r}}^j\right) \approx {\mathcal {J}}^{mn}\left( {\mathbf {x}};\,{\mathbf {r}}^j\right) = \sum _{p,q{:}p+q=0}^{p+q=P} D^{mn}_{pq} \left( x_1-r_1^j\right) ^p \left( x_2-r_2^j\right) ^q, \end{aligned}$$

for non-negative integers $p,\,q$ and some positive integer *P*,  where the notation ${\mathcal {J}}^{mn}$ has been used to indicate that this is an *approximation* to $J^{mn}.$ We will see in fact that this approach is useful for many practical cases, especially with an appropriate choice of ${\mathbf {r}}^j.$ Note that the upper limit $P\rightarrow \infty$ in general.

For this local expansion to be useful in (2.24), we need to re-introduce the dimensional variables, so that (3.7) becomes

3.8 $$\begin{aligned} \tilde{{\mathcal {J}}}^{mn}\left( \tilde{{\mathbf {x}}};\,\tilde{{\mathbf {r}}}^j\right) = \sum _{p,q{:}p+q=0}^{p+q=P} \tilde{D}^{mn}_{pq} \left( \tilde{x}_1-\tilde{r}_1^j\right) ^p \left( \tilde{x}_2-\tilde{r}_2^j\right) ^q, \end{aligned}$$

where $$\tilde{D}^{mn}_{pq}=D^{mn}_{pq}/L_j^{p+q}.$$

In order to evaluate the coefficients $D_{pq}^{mn}$ it is then useful to write (3.7) in terms of the orthogonal functions $\cos (n\theta ),\, \sin (n\theta ){\text {:}}$

3.9 $$\begin{aligned} {\mathcal {J}}^{mn}(\rho ,\,\theta ) = a^{mn}_0(\rho ) + \sum _{j=1}^{N} a_j^{mn}(\rho )\cos (j\theta ) + b_j^{mn}(\rho )\sin (j\theta ), \end{aligned}$$

where the coefficients $a_j^{mn},\, b_j^{mn}$ are determined explicitly in terms of $\rho$ and $D^{mn}_{pq}$ by employing the plane polar form (3.2)$_{I}$ for ${\mathbf {x}}-{\mathbf {r}}^j$ and then expand the powers of the resulting trigonometric functions in their multiple angle forms (this is straightforwardly done algorithmically in a symbolic package such as mathematica) so that for example

3.10 $$\begin{aligned} a_0^{mn}(\rho ) = D^{mn}_{00} + \frac{1}{2}\rho ^2\left( D^{mn}_{20}+D^{mn}_{02}\right) + \frac{1}{8}\rho ^4\left( 3D^{mn}_{40}+D^{mn}_{22}+3D^{mn}_{04}\right) +\cdots , \end{aligned}$$

and analogously for $a_j^{mn},\, b_j^{mn}.$

Next note that the $D_{pq}^{mn}$ are not independent. We can see this by applying the Laplacian operator to (3.7) and exploiting the form of (3.6) with $\nabla ^2 G + \delta (\mathbf {y}-{\mathbf {x}})=0$ to find

3.11 $$\begin{aligned} x_1^{m} x_2^{n}&= \sum _{p,q{:}p+q=2}^{p+q=P} D_{pq}^{mn}\left( p(p-1)x_1^{p-2}y_2^q+q(q-1)x_1^p x_2^{q-2}\right) . \end{aligned}$$

Comparing coefficients of $x_1^a x_2^b$ provides equations linking the coefficients $D_{pq}^{\delta \xi },$ so that for a fixed pair $m,\,n$ we have for $p,\,q\ge 0$

3.12 $$\begin{aligned} (p+2)(p+1)D_{(p+2)q}^{mn}+(q+2)(q+1)D_{p(q+2)}^{mn} = \delta _{pm}\delta _{qn}. \end{aligned}$$

Next introduce the operators ${\mathcal {C}}_k(*)$ and ${\mathcal {S}}_k(*)$ as those that multiply $*$ by $\cos (k\theta )$ and $\sin (k\theta ),$ respectively and then integrate over $\theta \in [0,\,2\pi ).$ Equate (3.3) and (3.9) and apply the operators ${\mathcal {C}}_m$ and ${\mathcal {S}}_m$ in turn to each side of the resulting equations to yield

3.13 $$\begin{aligned}&{\mathcal {C}}_k\left( J^{mn}\right) =\left( 1+\delta _{k0}\right) \pi a^{\delta \xi }_m(\rho ), \quad {\mathcal {S}}_k\left( J^{mn}\right) = \pi b^{\delta \xi }_m(\rho ), \end{aligned}$$

where $\delta _{m0}$ is the Kronecker delta. Without loss of generality, set $\rho =1,$ to find that

3.14 $$\begin{aligned}&2\pi a_0^{mn} = 2\pi a_0^{mn}(1) = \frac{1}{4\pi }\int _{0}^{2\pi }\int _0^{f_j(\theta )} \zeta ^{m+n+1}\cos ^{m}\phi \sin ^{n}\phi C_0(\zeta ,\,\phi ) \mathrm{d}\zeta \mathrm{d}\phi , \end{aligned}$$

3.15 $$\begin{aligned}&\pi a_k^{mn} = \pi a_k^{mn}(1) = \frac{1}{4\pi }\int _{0}^{2\pi }\int _0^{f_j(\theta )} \zeta ^{m+n+1}\cos ^{m}\phi \sin ^{n}\phi C_k(\zeta ,\,\phi ) \mathrm{d}\zeta \mathrm{d}\phi , \end{aligned}$$

3.16 $$\begin{aligned}&\pi b_k^{mn} = \pi b_k^{mn}(1) = \frac{1}{4\pi }\int _{0}^{2\pi }\int _0^{f_j(\theta )} \zeta ^{m+n+1}\cos ^{m}\phi \sin ^{n}\phi S_k(\zeta ,\,\phi ) \mathrm{d}\zeta \mathrm{d}\phi , \end{aligned}$$

where

3.17 $$\begin{aligned} C_k(\zeta ,\,\phi )&= \int _0^{2\pi }\cos (k\theta )\ln \left( \zeta ^2+1-2\zeta \cos (\theta -\phi )\right) \mathrm{d}\theta , \end{aligned}$$

3.18 $$\begin{aligned} S_k(\zeta ,\,\phi )&= \int _0^{2\pi }\sin (k\theta )\ln \left( \zeta ^2+1-2\zeta \cos (\theta -\phi )\right) \mathrm{d}\theta . \end{aligned}$$

Note again here that by construction, $\zeta =f_j(\phi )$ prescribes the boundary of the fibre with $f_j(\phi )\ge 1$ so that $\zeta \ge 1.$ By employing some exact results for certain integrals, we now show that we are able to write (3.14)–(3.16) in terms of a single integral in $\phi$ involving the boundary function $f_j(\phi ).$ To proceed, define

3.19 $$\begin{aligned} \psi&= \theta -\phi , \end{aligned}$$

and this substitution allows us to simplify the expression with various terms reducing to zero by employing double angle formulae for the trigonometric functions. We then obtain

3.20 $$\begin{aligned} C_k(\zeta ,\,\phi )&= \cos (k\phi ){\mathcal {I}}_k(\zeta ), \end{aligned}$$

3.21 $$\begin{aligned} S_k(\zeta ,\,\phi )&= \sin (k\phi ){\mathcal {I}}_k(\zeta ), \end{aligned}$$

where

3.22 $$\begin{aligned} {\mathcal {I}}_k(\zeta ) = \int _0^{2\pi } \cos (k\psi )\ln \left( \zeta ^2+1-2\zeta \cos \psi \right) \mathrm{d}\psi . \end{aligned}$$

Next we use the results (Gradshteyn and Ryzhik [34], Eq. 15 of Sect.  4.224 and Eq. 6 of Sect. 4.397)

3.23 $$\begin{aligned} {\mathcal {I}}_0(\zeta ) = \left\{ \begin{array}{l@{\quad }l} 0, &{} \zeta ^2<1, \\ 2\pi \ln \zeta ^2, &{} \zeta ^2>1, \end{array} \quad {\mathcal {I}}_k(\zeta ) = \left\{ \begin{array}{l@{\quad }l} -\frac{2\pi }{k}\zeta ^k, &{} \zeta ^2 <1, \\ -\frac{2\pi }{k}\zeta ^{-k}, &{} \zeta ^2>1, \end{array}\right. \right. \end{aligned}$$

where $k\ge 1$ in the latter. The $\zeta$ integration can then be straightforwardly carried through using integration by parts, to determine that

3.24 $$\begin{aligned} 2\pi a_0^{mn}&= \frac{1}{4}\int _0^{2\pi }\int _0^{f(\hat{\theta })}\zeta ^{m+n+1}\cos ^{m}\hat{\theta }\sin ^{n}\hat{\theta }{\mathcal {I}}_0(\zeta ) \mathrm{d}\zeta \mathrm{d}\hat{\theta }, \end{aligned}$$

3.25 $$\begin{aligned}&= \frac{1}{(m+n+2)^2}\int _0^{2\pi }\cos ^{m}\hat{\theta }\sin ^{n}\hat{\theta }\left[ (f(\hat{\theta }))^{m+n+2}((m+n+2)\ln f(\hat{\theta }) -1)+1)\right] \mathrm{d}\hat{\theta } \end{aligned}$$

3.26 $$\begin{aligned}&= F_0^{mn}, \end{aligned}$$

noting that we have introduced the notation $F_0^{mn}$ for this integral form of $a_0^{mn}$ so that we can refer to it later on. Proceeding analogously, defining

3.27 $$\begin{aligned} F^{mn}_{(A,k)}&= {-}\int _0^{2\pi }\cos ^{m}\phi \sin ^{n}\phi (A\cos (k\phi )+(1-A)\sin (k\phi ))\left[ \frac{1}{m+n+k+2} + {\mathcal {J}}_{m+n-k}(\phi )\right] \mathrm{d}\phi , \end{aligned}$$

where

$$\begin{aligned} {\mathcal {J}}_{m+n-k}(\phi ) = \left\{ \begin{array}{l@{\quad }l} \ln (f(\phi )), &{} m+n-k = {-}2, \\ \dfrac{(f(\phi ))^{m+n-k+2}-1}{m+n-k+2} , &{} \text{ otherwise }, \end{array}\right. \end{aligned}$$

it can be straightforwardly shown that, for $k\ge 1,$

3.28 $$\begin{aligned} a_0^{mn} = \frac{1}{2\pi }F_0^{mn}, \quad a_k^{mn} = \frac{1}{\pi }F_{(1,k)}^{mn}, \quad b_k^{mn} \frac{1}{\pi }F_{(0,k)}^{mn}. \end{aligned}$$

We now employ the equations relating $a_m,\, b_m$ and $D_{pq}$ and equate these to the integral forms just developed above, so that, e.g.

3.29 $$\begin{aligned} a_0^{mn}&= D_{00}^{mn} + \frac{1}{2}\left( D_{20}^{mn}+D_{02}^{mn}\right) + \frac{1}{8}\left( 3D_{40}^{mn}+D_{22}^{mn}+3D_{04}^{mn}\right) + \cdots = \frac{1}{2\pi }F_0^{mn}, \end{aligned}$$

3.30 $$\begin{aligned} a_1^{mn}&= D_{10}^{20} +\frac{1}{4}\left( D_{12}^{20}+3D_{30}^{20}\right) + \cdots = \frac{1}{\pi }F_{(1,1)}^{mn}, \end{aligned}$$

3.31 $$\begin{aligned} \cdots&= \cdots = \cdots , \end{aligned}$$

3.32 $$\begin{aligned} b_1^{mn}&= D_{01}^{20} +\frac{1}{4}\left( D_{21}^{20}+3D_{03}^{20}\right) = \frac{1}{\pi }F_{(0,1)}^{mn}, \end{aligned}$$

3.33 $$\begin{aligned} \cdots&= \cdots = \cdots \end{aligned}$$

These equations coupled with the conditions (3.12) appropriately truncated gives a closed system from which the coefficients $D_{pq}^{mn}$ are determined, i.e. this can be written

3.34 $$\begin{aligned} {\mathbf {A}}{\mathbf {D}}&= {\mathbf {F}}, \end{aligned}$$

where ${\mathbf {D}}$ is a vector of the unknowns $D_{pq}^{mn}$ and ${\mathbf {F}}$ is a vector of the right-hand sides associated with (3.29)–(3.33) and (3.12). It should be noted that the matrix ${\mathbf {A}}$ is identical regardless of the shape of the inhomogeneity and the expansion point ${\mathbf {r}}.$ The latter affect only the right-hand side ${\mathbf {F}}.$ Therefore one can immediately write the explicit form

3.35 $$\begin{aligned} {\mathbf {D}}&= {\mathbf {A}}^{-1}{\mathbf {F}}, \end{aligned}$$

and the only computation in order to form the polynomial approximation are the integrals in ${\mathbf {F}}.$ In the next section several examples are considered with various eigenstrains and a number of inhomogeneities of distinct shape, in order to illustrate the efficacy of this scheme.

## Method implementation

The aim then is to determine $\tilde{S}_{ij}^{mn}$ (2.20) via polynomial representations of the integral defined in (2.19) for a given domain $\tilde{D},$ integers $m,\,n$ and position vector $\tilde{\mathbf {s}}.$ We shall always choose $\min f(\theta ) = 1$ so that in terms of the original dimensional coordinates ${\mathcal {L}}=1.$ This means in effect that there is no difference between variables that have a ‘tilde’ and those that do not above. We shall talk in terms of variables without the tilde for convenience.

### Elliptical inclusions: polynomial conservation

**Fig. 4:**
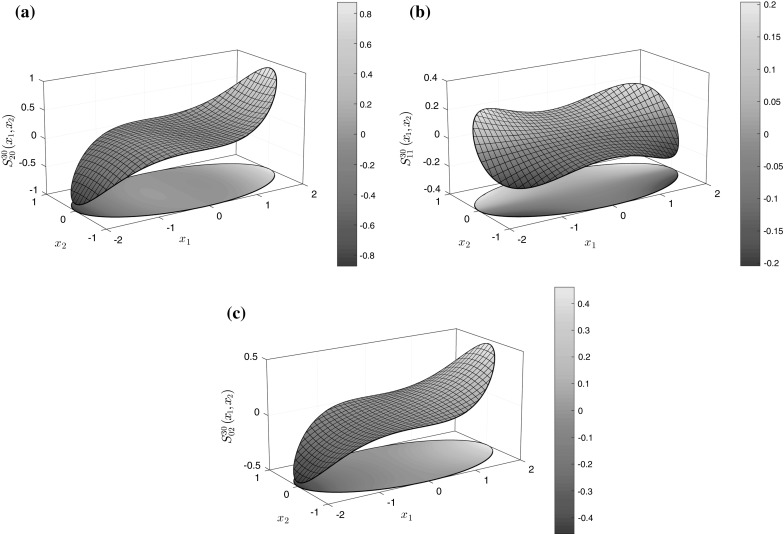
The components of the generalised Eshelby tensor for an ellipse of major axis 2 in the $x_{1}$ direction and minor axis 1 in the $x_{2}$ direction. (Color figure online)

First consider the case when $\mathbf {s}=\mathbf {0}$ and the inhomogeneity is elliptical, with

$$\begin{aligned} f(\theta ) = \frac{a_1 a_2}{\sqrt{a_1^{2}\sin ^{2}{\theta }+a_2^{2}\cos ^{2}{\theta }}}, \end{aligned}$$

where $a_1$ and $a_2$ are the semi-axes of the ellipse in the $x_{1}$ and $x_{2}$ directions, respectively. Note that for convenience, we choose $\min (a_1,\,a_2)=1.$ As stated above, for elliptical inhomogeneities, polynomial eigenstrains of order $m+n$ will give rise to $J^{mn}$ integrals that are polynomials of order $m+n+2.$ Our first check is therefore to ensure that the method above reproduces this so-called property of *polynomial conservation*. Let us compute $J^{30}$ which corresponds to a cubic eigenstrain. The method yields $J^{30}$ as a fifth-order polynomial and gives rise to the three components of the Eshelby tensor $S_{20}^{30},\, S_{11}^{30}$ and $S_{02}^{30}.$ With all coefficients taken to five significant figures, for $a_1=2,\, a_2=1,$ these polynomials take the form

$$\begin{aligned}&S_{20}^{30} = {-}0.098765 x_{1} + 0.13374 x_{1}^{3} +0.098765 x_{1}x_{2}^{2}, \\&S_{11}^{30}=0.098765 x_{2} + 0.098765 x_{1}^{2}x_{2}- 0.032922 x_{2}^{3}, \\&S_{02}^{30}= 0.098765 x_{1} + 0.032922 x_{1}^{3} -0.098765 x_{1} x_{2}^{2}, \end{aligned}$$

and surface plots of these components are provided in Fig. 4.

### Squares, pentagons and hexagons

Now consider domains of more complex form than elliptical and for now take $\mathbf {s}=\mathbf {0}.$ Gielis [35] utilised what is referred to as the *superellipse* shape function to reproduce many shapes commonly occurring in nature. The function is defined as

4.1 $$\begin{aligned} f(\theta ) = \left( \left| \frac{1}{a}\cos {\left( \frac{\gamma }{4}\theta \right) }\right| ^{\delta _{2}}+\left| \frac{1}{b}\sin {\left( \frac{\gamma }{4}\theta \right) }\right| ^{\delta _{3}}\right) ^{-1/\delta _{1}}. \end{aligned}$$

Here $\gamma$ can be considered a parameter associated with rotational symmetry, so for a given $\gamma$ the shape produced will have $\gamma$-fold rotational symmetry. Further, *a* and *b* are associated with the aspect ratio of the shape, and $\delta _{1},\, \delta _{2}$ and $\delta _{3}$ help to further control the form of the shape. For instance, to reproduce an asymmetric shape, $\delta _{1},\, \delta _{2}$ and $\delta _{3}$ should be chosen to have distinct values and to produce ‘star’ shapes, $\delta _{1}$ should be chosen less than $\delta _{2}$ and $\delta _{3}.$ Note that choosing $\delta _{1}=\delta _{2}=\delta _{3}=2$ and $\gamma =4$ returns the shape function governing an ellipse, with $a=b$ further reducing the equation to that of a circle or radius *a*. The function (4.1) is incredibly versatile, and can be used to produce close approximations to many boundary shapes, including polygons, with the essential rule of thumb being to choose $a=b=1,\, \gamma$ to be the degree of polygon sought, $\delta _{2}$ and $\delta _{3}$ to be large and equal, and $\delta _{1}$ to be larger still—except for the square case, where all three $\delta _j$ parameters are chosen to be equally large.

**Fig. 5:**
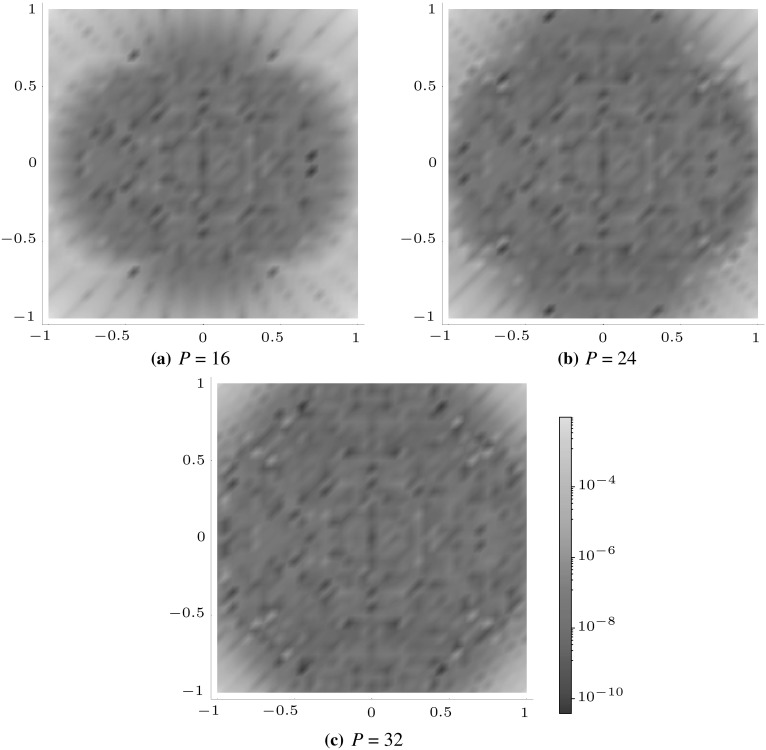
Logarithmic density plot of the error $J_\mathrm{e}^{20}$ as defined in (4.2) within a square domain ($\delta _{1} = \delta = 400$ and $\gamma =4$), where *P* is the order of the polynomial approximation. (Color figure online)

**Fig. 6:**
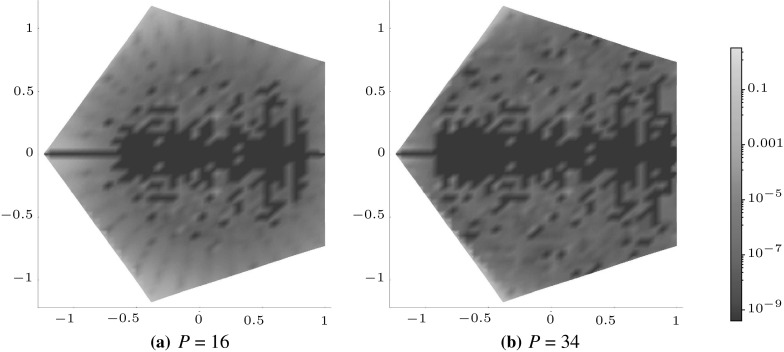
Logarithmic density plot of the error $J_\mathrm{e}^{03}$ as defined in (4.2) within a pentagonal domain ($\delta =310,\, \delta _{1} = 500$ and $\gamma =5$), where *P* is the order of the polynomial approximation. (Color figure online)

**Fig. 7:**
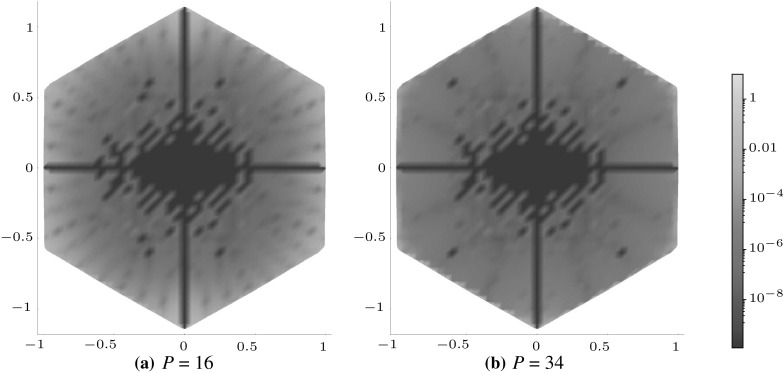
Logarithmic density plot of the error $J_\mathrm{e}^{11}$ as defined in (4.2) within a hexagonal inclusion ($\delta =40,\, \delta _{1} = 100$ and $\gamma =6$), where *P* is the order of the polynomial approximation. (Color figure online)

Figures 5, 6 and 7 illustrate how the polynomial approximation ${\mathcal {J}}^{mn}$ for square (with $m=2,\,n=0$), pentagonal (with $m=0,\, n=3$) and hexagonal (with $m=1,\, n=1$) inclusions tends to the numerical evaluation of the integral $J^{mn}$ as the order of the polynomial increases by plotting the following measure of the error,

4.2 $$\begin{aligned} J_\mathrm{e}^{mn}=\left| \frac{J^{mn}-{\mathcal {J}}^{mn}}{J^{mn}}\right| , \end{aligned}$$

across the whole of the domain. In each example $a=b=1$ and $\delta _{2}=\delta _{3}=\delta$ have been used in the superellipse definition, with the choices of the other parameters listed in the appropriate captions of the figures. Note that each shape features a different choice of *m* and *n* in order to illustrate that the scheme works equally well regardless of this choice.

These plots illustrate that as the order of polynomial approximation *P* increases, the blue regions (representing very small error) become more prominent and the yellow regions (representing moderate errors) become less so. In the case of the pentagon, both the polynomial approximation and the numerical evaluation are exactly zero when $x_{2}=0$ (due to the choice of *m* and *n*), hence the dark blue line in the direction of this axis. The same is true for the hexagonal case when $x_{1}=0$ and $x_{2}=0.$ Note that if better representations are required in regions of moderate error, the polynomial expansion could be taken about a point close to this location in order to obtain ‘patched’ expansions in different regions of space.

### A first case when ${\mathbf {r}}\ne \mathbf {0}{\text {:}}$ the limacon

So far no “re-expansion” has been required in the sense that the natural “origin” of the domains considered was the origin itself. Let us now consider the case of the so-called *limacon*, whose boundary is governed by the polar function

4.3 $$\begin{aligned} f(\theta ) = b+a\cos {\theta }. \end{aligned}$$

**Fig. 8:**
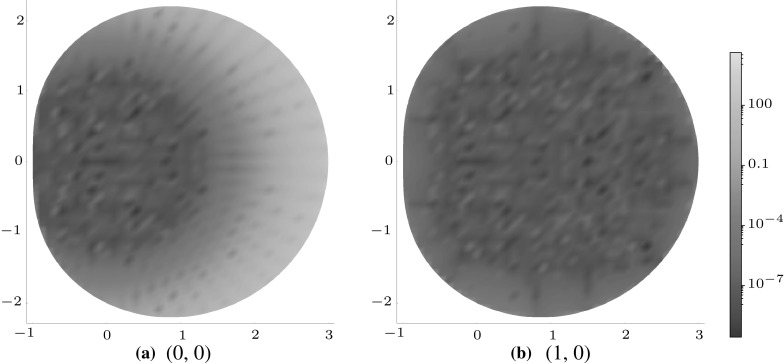
Logarithmic density plot of the error $J_\mathrm{e}^{02},$ as defined in (4.2), within a limacon inclusion ($a=1,\, b=2$), where ${\mathcal {J}}^{02}$ is the 24th-order polynomial approximation when basing the coordinate system at **a** the origin and **b** at the point (1, 0). (Color figure online)

Figure 8a considers the error in approximating $J^{02}$ for this shape with $a=1$ and $b=2$ by a 24th-order polynomial approximation with no re-expansion, i.e. ${\mathbf {r}}=\mathbf {0}.$ Notably, the error is particularly significant (bordering on order of magnitude 1000) in the farthest right-hand regions of the inclusion. This suggests that the centre of re-expansion should be positioned further to the right. Furthermore, the symmetry of the inclusion suggests the expansion point should remain along the $x_{1}$ axis. In fact, the most obvious way of maximising the radius of convergence would be to move the basis to ${\mathbf {r}}=(1,\,0),$ as a circle of radius 2 based at this point will not only fit inside the limacon, but also cover the vast majority of the area of this domain. Figure 8b illustrates how the error improves when this re-expansion point is chosen, with the regions previously featuring the most extreme errors now featuring errors in the region of $10^{-7}.$

### The ‘hourglass’ and hypogenis shapes

**Fig. 9:**
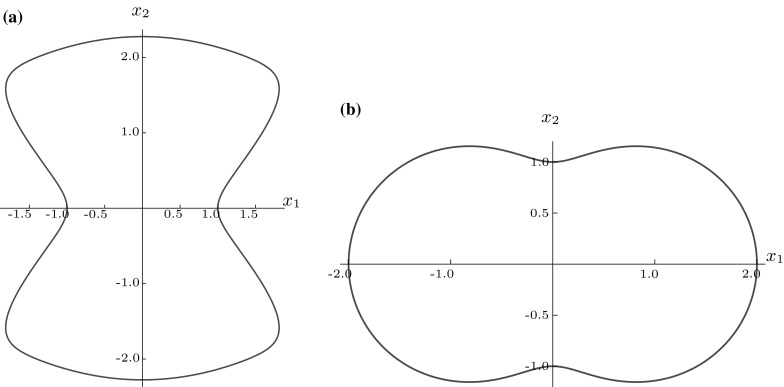
**a**‘Hourglass’ and **b** hypogenis-shaped inclusion

Let us now consider some more complex shapes of non-polygonal form. By choosing $\gamma =4,\, a=1,\, b=10,\, \delta _{1}=4.2,\, \delta _{2}=17$ and $\delta _{3}=1.5$ in the superellipse expression (4.1), we obtained what we shall term the ‘hourglass’ due to its resemblance to the traditional time-keeping device, as can be seen in Fig. 9a and take $\mathbf {s}=\mathbf {0}.$

**Fig. 10:**
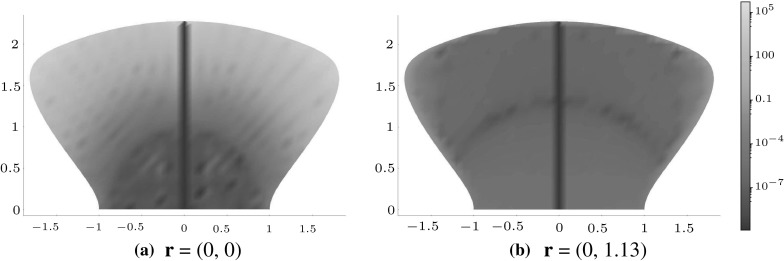
Logarithmic density plot of the error $J_\mathrm{e}^{12},$ as defined in (4.2), within an ‘hourglass’ domain ($x_2>0$ is shown here), where ${\mathcal {J}}^{12}$ is the 28th-order polynomial approximation while expanding the polynomial about **a** ${\mathbf {r}}=\mathbf {0}$ and **b** at the point ${\mathbf {r}}=(0,\,1.13),$. (Color figure online)

As one would expect, basing an expansion of the polynomial approximation at the origin yields large errors in the extreme north and south regions of the domain, as illustrated in Fig. 10a, focusing on the $x_{2}\ge 0$ region and illustrated in the case of $J^{12}.$ The symmetry of the domain is such that it makes sense to split it into two domains: $x_{2}\ge 0$ and $x_{2} < 0,$ and use expansions about two points on the $x_{2}$ axis equidistant from the origin in each. Once again, focusing on $x_{2}\ge 0,$ Fig. 10b shows how the error improves upon centering the expansion about ${\mathbf {r}}=(0,\,1.13).$

**Fig. 11:**
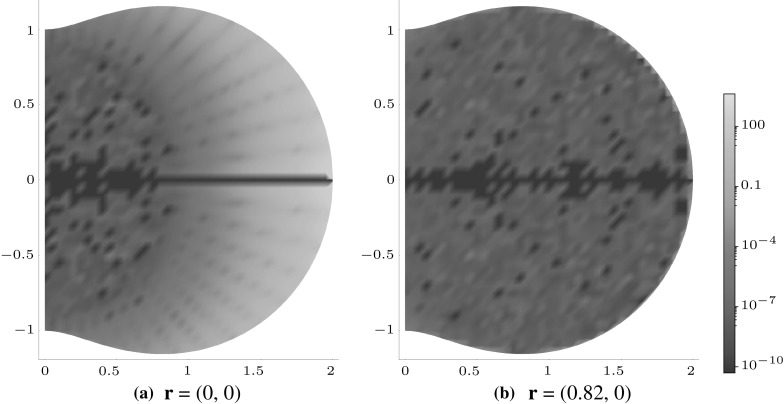
Logarithmic density plot of the error $J_\mathrm{e}^{21},$ as defined in (4.2), within a hypogenis inclusion, where ${\mathcal {J}}^{21}$ is the 28th-order polynomial approximation when expanding the polynomial about **a** ${\mathbf {r}}=\mathbf {0}$ and **b** at the point ${\mathbf {r}}=(0.82,\,0)$. (Color figure online)

An alternative to consider is the hypogenis, as depicted in Fig. 9b, boundary of which is described by

$$\begin{aligned} f(\theta ) = \frac{3}{2}\sqrt{\frac{1}{9}\sin ^{2}{2\theta }+\left( 1+\frac{1}{3}\cos {2\theta }\right) ^{2}}, \end{aligned}$$

with the factor of 3/2 giving rise to $f(\theta ) \ge 1.$ In a similar vein to the ‘hourglass’, here it is advantageous to split the domain into the sub-domains $x_{1}\ge 0$ and $x_{1} < 0$ and use expansions about two points on the $x_{1}$ axis equidistant from the origin for each. Figure 11 focuses on the $x_{1}\ge 0$ region and shows that an expansion about ${\mathbf {r}}=(0.82,\,0)$ improves upon an expansion about the origin.

### An eigenstrain case when $\mathbf {s}\ne \mathbf {0}$

**Fig. 12:**
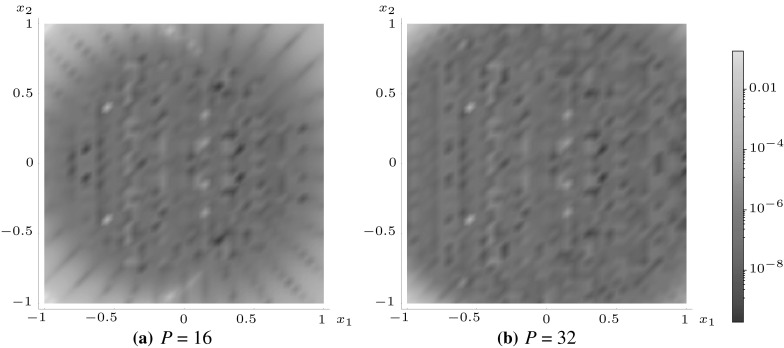
Logarithmic density plot of the error $J_\mathrm{e}^{20}$ as defined in (4.2) within a square inclusion, where *P* is the order of the polynomial approximation and $\mathbf {s}=(1,\,0)$. (Color figure online)

In all previous examples, $\mathbf {s}=\mathbf {0}$ and expansions were taken based on an appropriate choice of ${\mathbf {r}}$ when it would improve the convergence of the scheme in various subdomains of the inhomogeneity or inclusion region. Let us now consider an example case where $\mathbf {s} = (1,\,0),$ but the inclusion shape remains centred at the origin. Here the domain to be examined is once again a square. In the case of, for instance, $J^{20}({\mathbf {x}};\,(1,\,0)),$ the expansion formula (2.23) will need to be utilised for ${\mathbf {r}}=\mathbf {0}$ in order to express $J^{20}({\mathbf {x}};\,(1,\,0))$ in terms of $J^{mn}({\mathbf {x}};\,\mathbf {0})$ integrals—terms which themselves shall need to be approximated by polynomial expansions about the origin. Figure 12 illustrates how increasing the order of these expansions improves the overall accuracy of the approximation of $J^{20}({\mathbf {x}};\,(1,\,0)).$

**Fig. 13:**
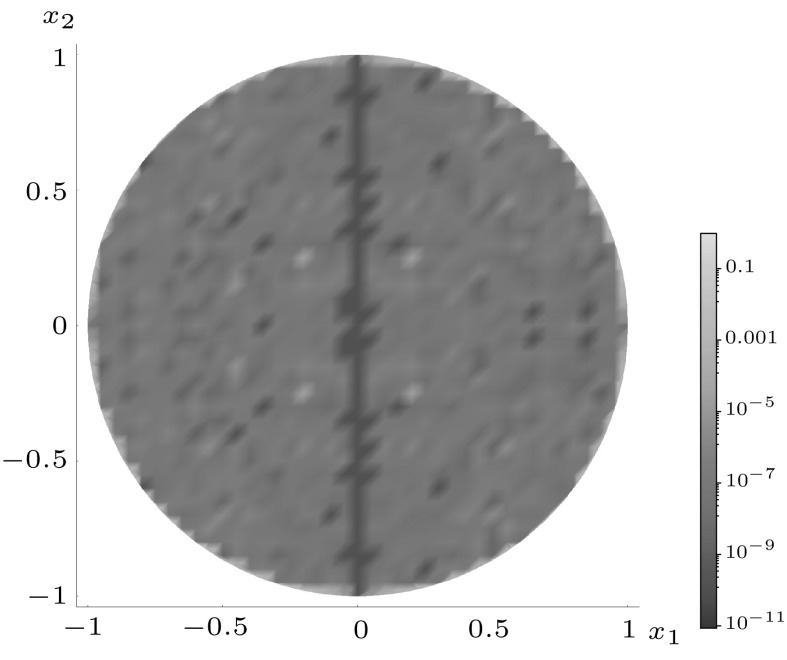
Logarithmic density plot of the error ${\mathcal {I}}^\mathrm{e}_{11,0}$ within a circular inclusion for eigenstrain $f({\mathbf {y}})=\sin {y_{1}}$. (Color figure online)

### Other eigenstrains

Finally, in order to illustrate the principle outlined in Sect. 2.4, (2.26) shall be examined for the case of a non-polynomial eigenstrain, e.g. when $f(\mathbf {y})=\sin {y_{1}}$ and in the case of a circular domain *D*. In this case, the eigenstrain may be expanded about $\mathbf {y}=\mathbf {0}$ to generate a sum of polynomial eigenstrain terms. The error in approximating the integral (2.26) by the truncated series (2.29) is defined as

4.4 $$\begin{aligned} {\mathcal {I}}^\mathrm{e}_{M,N}=\left| \frac{{\mathcal {I}}-{\mathcal {I}}_{M,N}}{{\mathcal {I}}}\right| . \end{aligned}$$

Figure 13 shows the error (4.4) when the Taylor expansion of the eigenstrain is truncated to the eleventh order ($M=11,\, N=0$).

## Concluding remarks

A new scheme to determine polynomial approximations to integrals over non-elliptical two-dimensional domains has been developed. We call these integrals *generalised Eshelby tensors* for the Newtonian potential problem since they correspond to taking non-uniform eigenstrains over the domain, and when the eigenstrain is uniform, this corresponds to the classical Eshelby tensor. Excellent performance of the method is illustrated by comparison with the numerical evaluation of the integral, noting that this has to be evaluated for every point in the domain of interest, whereas once determined, the polynomial approximation can be rapidly evaluated at any point. Extensions to the two-dimensional in-plane elasticity case are underway and also to the full three-dimensional scalar and tensor scenarios. Further investigation is also underway that will explain theoretically the domains of convergence of the polynomial expansions.
